# Impact of a month-long training program on the clinical skills of ophthalmology residents and practitioners

**DOI:** 10.4103/0301-4738.64141

**Published:** 2010

**Authors:** Sushma Tejwani, Somasheila I Murthy, Chandra Sekhar Gadudadri, Ravi Thomas, Praveen Nirmalan

**Affiliations:** Department of Ophthalmology, L. V. Prasad Eye Institute, Kallam Anji Reddy Campus, Hyderabad, India; 1 International Center for Advancement of Rural Eye Care, L.V Prasad Eye Institute, Kismatpur Campus, Hyderabad, India

**Keywords:** Evaluation of training program, medical training, ophthalmology residency program, subjective assessment

## Abstract

A cohort study was performed to assess the impact of an intensive, hands-on, supervised training program in ophthalmic clinical evaluation, for ophthalmology residents and private practitioners. All students underwent one-month training in comprehensive ophthalmology examination and investigations at a tertiary care center between January 2004 and January 2006. The training methodology included didactic lectures, video-demonstrations and hands-on training. The participants completed a self-assessment with a set of 23 questions designed to assess the level of confidence in various skills on the first and last day of the training. Of a total of 118 students, 67 (56.8%) were residents and 51 (43.2%) were practitioners. The mean score pre-training was 38.3 out of 92 (S.D. ±16.9), and was 70.6 out of 92 (S.D.± 10.1) post-training. The mean increase in the scores was 32.3 (*P* value < 0.001). We concluded that intensive, short-term training programs could improve the self-perceived level of confidence of ophthalmology residents and practitioners.

Ophthalmology has undergone significant changes in all aspects with several devices now available to aid in the examination and diagnosis of eye disorders, and these are now an integral part of eye care centers in the developed world. However, very few centers in developing countries can claim to be on par with their developed counterparts.[1]

In India, there are more than 110 medical colleges that offer ophthalmology postgraduate training programs.[2] Unfortunately, due to various factors, the majority of these programs may lack infrastructure, facilities, experienced faculty and sometimes even the most basic of diagnostic equipment.[2] We have multiple recent reports indicating the status of ophthalmic residency training in India.[3,4] It is quite possible that some of these graduates from these programs are unfamiliar with the basic routine eye examination techniques. To bridge this gap, a training program was initiated which offers intensive and supervised training in clinical and investigational methods in ophthalmic evaluation. The aim of the study was to assess the impact of an intensive, hands-on, supervised training program in ophthalmic clinical evaluation, for ophthalmology residents and private practitioners.

## Materials and Methods

**Figure 1:**
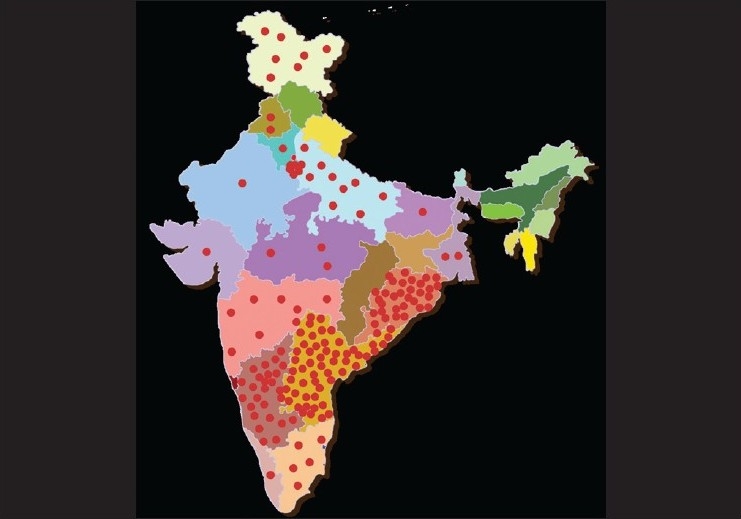
Number of dots on the country map represents the number of students from different parts of the country who were trained in this particular program

Ophthalmology residents from different medical colleges in India and a few other countries [Fig. 1] were the participants in this month-long training program. The students who received training from January 2004 to January 2006 at a tertiary care centre were included in the study. The institutional board was approached for approval and they had recommended that it was not required for the study. This course schedule included daily teaching by demonstration of various techniques on patient-volunteers in the clinics and video demonstrations and didactic lectures by the faculty. The program syllabus is listed in Table 1. In a given month, there were six to eight trainees who utilized three examination rooms. The course was divided into two halves:

**Table 1:** Syllabus of the one-month training program


Cornea
External examination
Slit-lamp evaluation techniques
Keratometry
A-scan biometry
Slit-lamp photography
Corneal scrapings
Dry eye evaluation
Glaucoma
Applanation tonometry
Gonioscopy
Slit-lamp binocular indirect ophthalmoscopy with 78D lens
Automated perimetry: interpretation and application
Retina
Indirect ophthalmoscopy
Slit-lamp binocular indirect ophthalmoscopy with 90 lens
Fundus fluorescein angiography and its interpretation
B-scan ultrasonography and its interpretation
Electrophysiology interpretation

1. Clinical methods teaching for the first two weeks
2. Diagnostic investigations for the next two weeks.

Clinical methods teaching: Sixty per cent of the time of the trainee was spent in the clinical area. The setup included three examination lanes, where the participants independently examined the patients under close supervision of a faculty member. The examination included history-taking, refraction, ocular motility and strabismus assessment, slit-lamp examination, applanation tonometry, gonioscopy (using four-mirror), stereoscopic slit-lamp biomicroscopy of the fundus with 90 diopter (D)/ 78D lens and indirect ophthalmoscopy. Additional clinical workup in special situations like infectious keratitis, dry eye evaluation, lacrimal drainage disorders, ptosis, squint and proptosis was also taught.

Advanced diagnostic investigations: In these labs, the trainees observed, learnt and assisted in various diagnostic procedures of each subspecialty such as cornea, glaucoma and retina. They had exposure to the working of corneal topography and A-scan biometry in cornea labs; automated perimetry (Humphrey visual field analyzer) in the glaucoma labs and B-scan ultrasonography (USG), fundus fluorescein angiography (FFA), electroretinography (ERG), and visual evoked potentials (VEP) in the retina labs.

Didactic lectures and video-demonstrations: Concurrent with the lab training, the course also included 25 didactic lectures and video-demonstrations. The subjects covered all areas of ophthalmology, like cornea, glaucoma, retina, oculoplasty, pediatric ophthalmology, clinical microbiology, pathology, contact lens, low vision and ocular radiology and documentation of clinical findings [Table 2]. In addition, reading material comprising a collection of peer-reviewed journal articles of interest and specially designed reviews of basic clinical skills and diagnostics for the students were also distributed to them.

**Table 2:** List of didactic lectures and videos


List of videos
Techniques of basic ocular examination
Guide to slit-lamp biomicroscopy
Evaluation of congenital ptosis
Exophthalmometry
Practical management of non-infectious keratitis
Gonioscopy: Learn and Teach
Goldmann Applanation Tonometry
Lab diagnosis of ocular infections
Corneal topography: Principles and applications
Ophthalmic ultrasound: B-scan Principles and applications
List of lectures
Cornea drawings
Evaluation of a case of infectious keratitis
Corneal topography and orbscan
Evaluation of a case of dry eye
Gonioscopy – Technique and interpretation
Disc evaluation
Humphrey visual fields analysis
Retina drawings
Fundus fluroscein angiography
Ultrasound B-scan
Basic evaluation and diagnosis of lacrimal disorders
Evaluation of a squint case
Interpretation of CT scan and MRI
A-scan and Biometry
Ocular pathology and microbiology

Self-assessment questionnaire: The participants were given a set of 23 questions [Table 3], which was essentially a self-reporting of the comfort level in performing a given diagnostic evaluation. The trainees answered the questionnaire on the first and last day of the course.

**Table 3:** Self-assessment questionnaire

Proforma for LVP-ZEISS trainees	
Name:	Date:
Period of Zeiss training:	
Qualifications and present status:	
Place:	
Scoring system	
Never done	0
Done occasionally	1
Done but not confident	2
Comfortable	3
Confident (can do in any situation)	4
Slit-lamp examination techniques	
Q.1. Diffuse illumination - 0 1 2 3 4	
Q.2. Direct focal - 0 1 2 3 4	
Q.3. Indirect focal - 0 1 2 3 4	
Q.4. Sclerotic scatter - 0 1 2 3 4	
Q.5. Retroillumination - 0 1 2 3 4	
Q.6. Specular reflection - 0 1 2 3 4	
Documentation of cornea findings with color codes	
Q.7. Comfort level - 0 1 2 3 4	
Keratometry, A-scan and IOL power calculation	
Q.8. Comfort level - 0 1 2 3 4	
Infectious keratitis workup	
Q.9. Comfort level - 0 1 2 3 4	
Dry eye evaluation	
Q.10. Comfort level - 0 1 2 3 4	
Gonioscopy	
Q.11. Comfort level - 0 1 2 3 4	
Q.12. Four-mirror gonioscopy Comfort level - 0 1 2 3 4	
Applanation tonometry	
Q.13. Comfort level - 0 1 2 3 4	
Documentation of gonioscopic findings	
Q.14. Comfort level -0 1 2 3 4	
Interpretation of Humphrey visual fields	
Q.15. Comfort level - 0 1 2 3 4	
Slit-lamp biomicroscopy of disc and macula (78D\90D)	
Q.16. Comfort level - 0 1 2 3 4	
Indirect ophthalmoscopy (+20D)	
Q.17. Comfort level - 0 1 2 3 4	
Documentation of retina findings with color codes	
Q.18. Comfort level - 0 1 2 3 4	
Fundus fluorescein angiography	
Q.19. Interpretation - 0 1 2 3 4	
Interpretation of B-scan findings	
Q.20. Comfort level - 0 1 2 3 4	
Interpretation of Amsler test and color vision	
Q.21. Comfort level - 0 1 2 3 4	
Electrophysiology (Interpretation)	
Q.22. Comfort level - 0 1 2 3 4	
Interpretation of CT scan and MRI	
Q.23. Comfort level - 0 1 2 3 4	

The maximum possible score for a trainee was 92 and minimum was 0. The maximum score per question was 4. The pre-training and post-training scores were compared and the results were analyzed to understand the impact of the training. Statistical analysis was done by paired t test using 11^th^ version of Software package for social sciences (SPSS).

## Results

**Figure 2:**
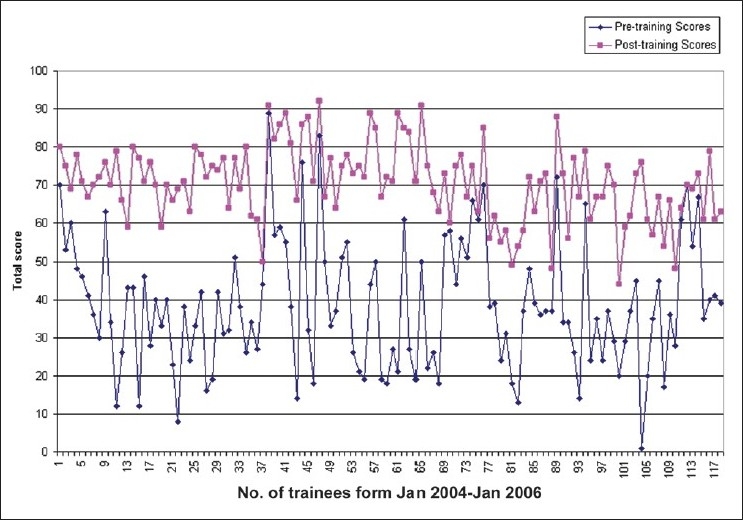
Comparison of scores before and after training of 118 subjects

Between January 2004 and January 2006, 137 trainees participated in the program. Of these, 118 completed all the days of the course and the questionnaires of these participants are included in the analysis. The trainees came from different parts of India and from Ghana, Vietnam, Bangladesh, Sri Lanka, Kingdom of Saudi Arabia and Paraguay [Fig. 1]. Of the 118 trainees there were 67 (56.8%) residents who were in their first, second or third year of post-graduation, and 51 (43.2%) practicing ophthalmologists (who had completed their post-graduation one to 15 years prior). The mean pre-training and post-training scores are depicted in a line diagram in Fig. 2. At entry the mean score (of the self-assessment) was 38.3 out of 92 (S.D. ±16.9)) for the entire group which became 70.6 out of 92 (S.D.± 10.1) at the end of the one-month training [Table 4]. The mean increase in score was by 32.3 units. For individual questions, the mean difference was the highest for "specular reflection technique of slit-lamp examination" (2.39), followed by "sclerotic scatter method of slit-lamp examination" (2.09) and "four-mirror gonioscopy" (2.09). At the entry level, the mean scores were <1 (never done or occasionally done) for "specular reflection", "four-mirror gonioscopy", "documentation of retina findings", "interpretation for Amsler" and "interpretation of color vision tests"; all of which improved significantly post-training. The post-training scores of "all techniques of slit-lamp evaluation", "gonioscopy", "applanation tonometry", "visual field analysis" and "slit-lamp biomicroscopy" uniformly increased to >3 (comfortable, confident).

**Table 4:** Mean score of the students before and after the training program

Question No.	Mean pretraining score	Mean posttraining score	Mean difference	Confidence intervals	Significance (2-tailed)
1.	3.39	3.93	0.54	0.40-0.68	<0.001
2.	3.17	3.90	0.72	0.55-0.90	<0.001
3.	2.54	3.80	1.26	1.04-1.48	<0.001
4.	1.75	3.83	2.09	1.85-2.32	<0.001
5.	2.31	3.75	1.44	1.23-1.65	<0.001
6.	0.82	3.21	2.39	2.16-2.63	<0.001
7.	1.33	3.39	2.06	1.83-2.29	<0.001
8.	2.78	3.28	0.50	0.31-0.69	<0.001
9.	1.91	2.92	1.01	0.82-1.20	<0.001
10.	1.55	2.69	1.14	0.93-1.35	<0.001
11.	1.85	3.52	1.67	1.45-1.89	<0.001
12.	0.83	2.64	1.82	1.52-2.11	<0.001
13.	1.55	3.65	2.09	1.84-2.35	<0.001
14.	1.55	3.27	1.72	1.51-1.94	<0.001
15.	1.32	2.83	1.51	1.29-1.73	<0.001
16.	2.06	3.48	1.42	1.19-1.65	<0.001
17.	2.11	3.25	1.14	0.96-1.32	<0.001
18.	1.36	2.95	1.59	1.36-1.81	<0.001
19.	0.96	2.45	1.49	1.27-1.71	<0.001
20.	1.26	2.65	1.39	1.19-1.59	<0.001
21.	1.46	2.85	1.39	1.16-1.62	<0.001
22.	0.35	1.77	1.42	1.22-1.63	<0.001
23.	1.08	2.08	1.00	0.83-1.17	<0.001
Total	38.3	70.58	32.26	29.4-35.2	<0.001

The least increase in the mean scores was noted in Questions 1 (diffuse illumination technique of slit-lamp examination), 8 (keratometry, A-scan) and 2 (direct focal illumination), which was 0.50, 0.54 and 0.72 respectively. Overall, these were the only three questions out of twenty-three (8.6%) where the participants rated themselves as comfortable/confident at the entry level. This improved to 13/23 questions (56.5%) at the end of the training. Only one question (No. 22) i.e. electrophysiology at the end of the training had a mean score of less than 2, however, there was a significant improvement in this score as well (1.42), compared to the pre-training level. For computed tomography (CT) scan interpretation and microbial keratitis workup, the mean scores at entry levels were 1.08 and 1.91 respectively, and these scores improved by 1 unit. No significant difference was observed in the scores of the residents' self-assessments as compared to the practitioners.

## Discussion

Residency programs in ophthalmology all over the world have several limitations,[1] this is particularly so in India where the standards are variable and there is no governing body for ophthalmology education and training. Murthy *et al*.,[2] have reported the variable situation of residency programs in India. The present program was started with a view to fill the gap between advancements in ophthalmology and deficient residency programs. To our knowledge, this is the first such report of assessment of a short-term training program by the participants in India.

Since it is logistically difficult for residents to attend a training program for a longer period during their residency, the duration of this program is one month only. Although the duration is short, the training is intensive as it involves examining patients along with live demonstrations and supplementation by audio-visual training. It also involves interaction and discussion with fellowship-trained faculty members of the tertiary care center. In the previous years, this program had received a very positive subjective feedback from the participants. However, the exact impact was not measured so far in any way. In this article, we have attempted to assess the impact of the program by assessing the change in the confidence levels of the trainees. This would also help judge the strengths and weaknesses of the program to make it better. The next step is to objectively evaluate the students in the beginning and at the end of the program for the same parameters and observe the results. This prospective study is underway and the results would be available shortly.

There are only a few reports for assessment of clinical skills in ophthalmology residency; but these (OCAT,[5] OCEX[6]) are for three-or four-year residency programs. Lippa *et al*.,[7] reported a study where the subjective assessment of medical students was done. The authors reported that nearly 60% students had erosion in their clinical skills at the end of the third year and reinforcement of these skills in the fourth year improved their performance.

In our study, we have used a specially designed questionnaire which is answered by the participant on the first day of the program and then again on the last day. The entry scores, when compared between each participant show a wide variability [Fig. 2]. This is a reflection of the variability in the training received by the students at their medical colleges, which may range from poor to excellent.

In this study, there was a significant difference noted in the confidence levels of these trainees at entry and exit. At pre-training, the average score was 38.3, which rose to 70.6. The training program therefore improved the level of confidence by 32.3 units. The maximum benefit was seen for slit-lamp examination techniques, especially the sclerotic scatter and specular reflection. Our findings suggest that the residents who participated in our program were not familiar with these techniques before joining the training program. While this could imply perhaps that there may exist some lacunae in some of the resident training programs, it could equally mean that those residents who joined this program are from colleges where the facilities are not available or the residents are not confident in their skills. On the other hand, proficiency in A-scan biometry rated a higher score at entry (2.78 out of 4) suggesting that training in the medical colleges is good in this area. Other areas where only a single unit increase in confidence scores was noted were interpretation of CT scan, magnetic resonance imaging (MRI) scan and infectious keratitis workup. This implies that our current program should be modified to include more training in these areas and lesser time should be spent for A-scan, keratometry, diffuse illumination technique and direct focal technique of slit-lamp examination. This data obtained from the present study may provide preliminary information to education bodies which may redesign ophthalmology programs and reallocate resources.

The major limitation of the study is that the data obtained is subjective and relies on self-assessment by the students. The improvement in their level of confidence does not necessarily mean improvement in their actual performance, practice and diagnosis. This is therefore a measure of improvement in the level of confidence of the ophthalmologist. The objective evaluation of the trainees before and after the training program and ideally again after six or 12 months would perhaps better assess the real impact of the program.

To summarize, the present study was conducted to evaluate the program conducted at a tertiary care center in India. If the score is indeed indicative of the actual clinical performance such programs can perhaps be included in the regular curriculum of postgraduate ophthalmology training in the country. It is concluded that intensive, short-term training programs can improve the self-perceived level of confidence of ophthalmology residents and practitioners.
